# Brain Parenchyma (pons) Involvement by Visceral Leishmaniasis: A Case Report

**Published:** 2018

**Authors:** Moslem SEDAGHATTALAB, Arsalan AZIZI

**Affiliations:** 1. Dept. of Internal Medicine, Yasuj University of Medical Sciences, Yasuj, Iran; 2. Dept. of Pathology, Yasuj University of Medical Sciences, Yasuj, Iran

**Keywords:** Brain, Pons, Leishmaniasis, Visceral

## Abstract

Leishmaniasis, as a vector-borne disease, is transmitted by sandfly and caused by *Leishmania* protozoa. Brain involvement rarely occurs in visceral leishmaniasis. In this paper, a rare case of pons involvement by visceral leishmaniasis (VL) is reported. A 54 yr old man from Southwest of Iran (Yasuj) presented to the Emergency Ward with a 3-wk history of headache (continuous, throbbing, and general), fever, chills, weakness, anorexia, and weight loss.

## Introduction

Leishmaniasis is a vector-borne disease, transmitted by sandfly and caused by *Leishmania* protozoa. This parasite affects the human’s reticuloendothelial system. A wide range of syndromes is produced by Leishmania species, including visceral leishmaniasis (VL), mucosal leishmaniasis, and cutaneous leishmaniasis (1).

In Iran, *L. infantum* is the main etiological agent of human VL and *L. tropica* is the second cause of VL. The incidence of visceral and cutaneous leishmaniasis is 0.092 and 22 per 100000 population, respectively. Kala-azar is endemic in southern and northwestern areas of Iran but VL also has been reported sporadically in other parts of country. The main reservoir hosts for *L. infantum* are domestic dogs and wild canines in various parts of country (2–4).

Neurologic involvement occurs rarely in visceral leishmaniasis (5). In this paper, a man with visceral leishmaniasis is described, with headache and fever, and imaging revealed involvement of brain in pons area.

## Case presentation

A 54 yr old man from Southwest of Iran (Yasuj) presented to the Emergency Ward with a 3-wk history of headache (continuous, throbbing, and general), fever, chills, weakness, anorexia, and weight loss. He also had a history of benign prostatic hyperplasia, gastroesophageal reflux disease, and hemorrhoid. Medications were tamsulosin, propranolol, rabeprazole, and cathartic syrup. His parents had no any congenital or infectious diseases.

On examination, the body temperature and blood pressure were 38 °C and 130/82 mm Hg, respectively. Abdominal examination revealed mild tenderness in right upper quadrant and moderate splenomegaly. All other examinations were normal.

The hemoglobin was 8.1 (gr/dl), the white blood cell count 1900, retic count 0.5%, and the platelet count 20000. The ESR was 56 (mm/h), alanine aminotransferase 84 (Iu/l), aspartate aminotransferase 67 (Iu/l), alkaline phosphatase 401 (Iu/l), albumin 3.4 (gr/dl), conjugated bilirubin 0.6 (mg/dl), ferritin 658.6 (mcg/dl), serum iron 23 (mcg/dl), total iron binding capacity (TIBC) 116 (mcg/dl). Other laboratory tests such as creatinine, blood sugar, partial thromboplastin time, prothrombin time, urinalysis, stool exam, sputum exam, wright test, 2ME, calcium, phosphorus, magnesium, and prostate-specific antigen were normal. Serologic tests for HBV, HCV, and HIV were negative.

Abdominal ultrasonography revealed mild hepatomegaly and moderate splenomegaly. Doppler ultrasound of abdomen showed dilation of splenic veins such as superior mesenteric vein (15 mm) and portal vein (15 mm).

**Fig. 1:**
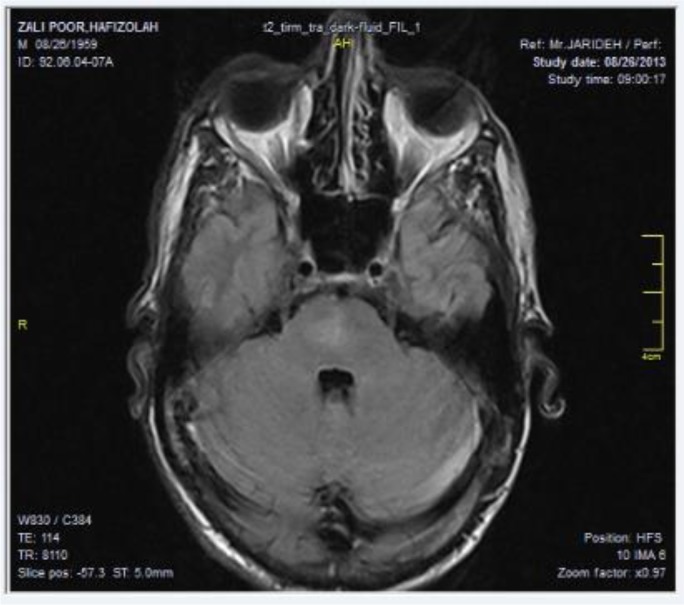
MRI image (FLAIR) revealed an increase in signal intensity of pons

**Fig. 2:**
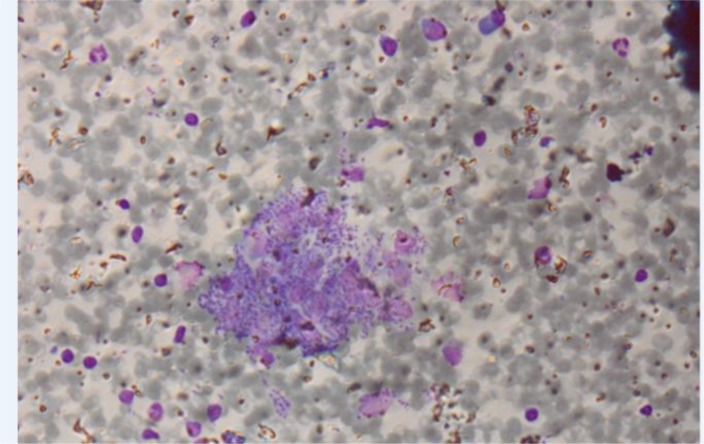
Bone marrow exam revealed macrophages with numerous *Leishmania* amastigotes (magnification: 400×)

A computerized tomography (CT) scan of brain revealed no abnormal finding. Axial fluid-attenuated inversion recovery MRI image (FLAIR) revealed an increase in signal intensity of central part of right side of pons (Fig. 1). Bone marrow aspiration and biopsy revealed macrophages with numerous *Leishmania* amastigotes (Fig. 2). Treatment with amphotericin B was initiated and the patient symptoms resolved completely. In second bone marrow exam, no amastigote was observed.

Five months after initial presentation and treatment with amphotericin B our patient has no any neurological morbidity and disability.

## Discussion

Visceral leishmaniasis (known as Kala-azar) is reported in Middle East countries, Pakistan, India, Sudan, Ethiopia, Kenya, Uganda, Somalia, Mediterranean Europe, Mexico, Argentina, and Brazil. Clinical manifestations of visceral leishmaniasis include fever, chills, weight loss, weakness, splenomegaly, and hepatomegaly. Serum levels of liver aminotransferases and immunoglobulins are raised, also they have anemia, thrombocytopenia, and leukopenia (1). In Iran, the main symptoms and signs in patients with Kala-azar included paleness, fever, splenomegaly, hepatomegaly, and lymphadenopathy (2).

In leishmaniasis, involvement of peripheral nervous system is more common than central nervous system (6). Peripheral nervous system involvement was reported as burning feet, foot drop, hearing loss, and multiple cranial nerve palsies (7). Schwann cells may act as a target for Leishmania (8).

We described a rare case of pons involvement by visceral leishmaniasis. Although involvement of brain parenchyma in visceral leishmaniasis is rare and mechanism of central and peripheral nervous system involvement by leishmaniasis is poorly understood, but haematogenous dissemination was described as mechanism of central nervous system involvement by visceral leishmaniasis (9).

## Conclusion

Leishmaniasis should be considered in differential diagnosis of patients who presented with fever, headache, and central nervous system lesions.
